# Population Genomic Structure and Recent Evolution of *Plasmodium knowlesi*, Peninsular Malaysia

**DOI:** 10.3201/eid2608.190864

**Published:** 2020-08

**Authors:** Suzanne E. Hocking, Paul C.S. Divis, Khamisah A. Kadir, Balbir Singh, David J. Conway

**Affiliations:** London School of Hygiene and Tropical Medicine Department of Infection Biology, London, UK (S.E. Hocking, D.J. Conway);; Universiti Sarawak Malaysia Malaria Research Centre, Kota Samarahan, Malaysia (P.C.S. Divis, K.A. Kadir, B. Singh, D.J. Conway)

**Keywords:** malaria, parasites, parasitic infections, mosquitoes, vector-borne diseases, mosquito-borne diseases, zoonoses, genomics, sequencing, population genetics, selection, epidemiology, transmission, *Plasmodium knowlesi*, Borneo, Malaysia, Peninsular Malaysia

## Abstract

Most malaria in Malaysia is caused by *Plasmodium knowlesi* parasites through zoonotic infection from macaque reservoir hosts. We obtained genome sequences from 28 clinical infections in Peninsular Malaysia to clarify the emerging parasite population structure and test for evidence of recent adaptation. The parasites all belonged to a major genetic population of *P. knowlesi* (cluster 3) with high genomewide divergence from populations occurring in Borneo (clusters 1 and 2). We also observed unexpected local genetic subdivision; most parasites belonged to 2 subpopulations sharing a high level of diversity except at particular genomic regions, the largest being a region of chromosome 12, which showed evidence of recent directional selection. Surprisingly, we observed a third subpopulation comprising *P. knowlesi* infections that were almost identical to each other throughout much of the genome, indicating separately maintained transmission and recent genetic isolation. Each subpopulation could evolve and present a broader health challenge in Asia.

All endemic human malaria parasite species originated as zoonotic crossover infections from nonhuman primates (*1*–*3*) and now cause approximately half a million human deaths annually (*4*). Until recently, zoonotic malaria was considered to be very rare, but original findings in Malaysia (*5*,*6*) and subsequent surveys elsewhere have revealed that many human malaria cases in Southeast Asia are caused by the macaque parasite *Plasmodium knowlesi* (*7*). This parasite species now causes almost all malaria in Malaysia (*4*) and is responsible for clinical cases throughout Southeast Asia, where the distributions of macaque reservoir hosts and mosquito vectors overlap with human populations (*8*). As several countries in Southeast Asia are working toward eliminating malaria, *P. knowlesi* represents a special public health challenge. Because of the presence of wild reservoir hosts, elimination of *P. knowlesi *is unlikely, and the problem will deepen if the parasite adapts or environments change to enable more effective transmission between humans (*9*). Of particular concern, numbers of cases each year are continuing to increase (*4*), and intensive surveillance in particular areas indicates this increase is not attributable to ascertainment bias (*10*).

Population genetic studies are essential to determining whether recent parasite adaptation has occurred, which might reflect ongoing evolution that is likely to affect the epidemiology. The *P. knowlesi* parasite has a ≈25 megabase genome of 14 chromosomes (*11*,*12*), haploid in blood stage infections and recombining in a brief diploid stage after male and female parasites mate in the mosquito vector, so informative studies require analysis of loci throughout the genome. Understanding of *P. knowlesi* population genetics has been gained by microsatellite genotyping (*13*,*14*) and whole-genome sequencing (*15*,*16*). In Malaysian Borneo, *P. knowlesi* consists of 2 genetically divergent populations (termed clusters 1 and 2) associated with different reservoir hosts: cluster 1 with long-tailed macaques (*Macaca fascicularis*) and cluster 2 with pig-tailed macaques (*M. nemestrina*) (*14*). In Peninsular Malaysia, on the mainland of Asia, a different genetic subpopulation of *P. knowlesi* exists. This subpopulation was initially indicated by comparing genome sequences of a few old laboratory isolates from Peninsular Malaysia with genome sequences of recent clinical samples from Borneo (*15*), and by comparing sequences of 2 genes in clinical samples from both areas (*17*). Subsequent multilocus microsatellite analysis of recent clinical cases of *P. knowlesi* infection from Peninsular Malaysia has confirmed that all cases are attributable to the cluster 3 type (*13*).

Experimental studies have been conducted on only a few strains of *P. knowlesi*, isolated many years ago and maintained in laboratory monkeys (*18*). Genome sequencing has revealed these strains to be of the cluster 3 type (*13**,**15*), and one of them has been adapted to in vitro culture in human erythrocytes, using 2 independent approaches involving culture with mixtures of macaque and human erythrocytes before growth in human erythrocytes alone (*19*,*20*). The short-term adaptability of this single strain is further illustrated by selection for culture in long-tailed macaque erythrocytes, which was associated with the loss of a specific ligand gene needed for invading human erythrocytes (*21*). These examples from laboratory observations strongly suggest that highly diverse natural parasite populations are likely to adapt to changing conditions.

All of the separately occurring *P. knowlesi* populations might evolve and emerge to present an even more serious public health challenge than already realized. To determine the population genetic substructure within *P. knowlesi* locally, we analyzed recent clinical samples from patients with *P. knowlesi* infection in Peninsular Malaysia by using whole-genome sequencing.

## Materials and Methods

**Figure 1:**
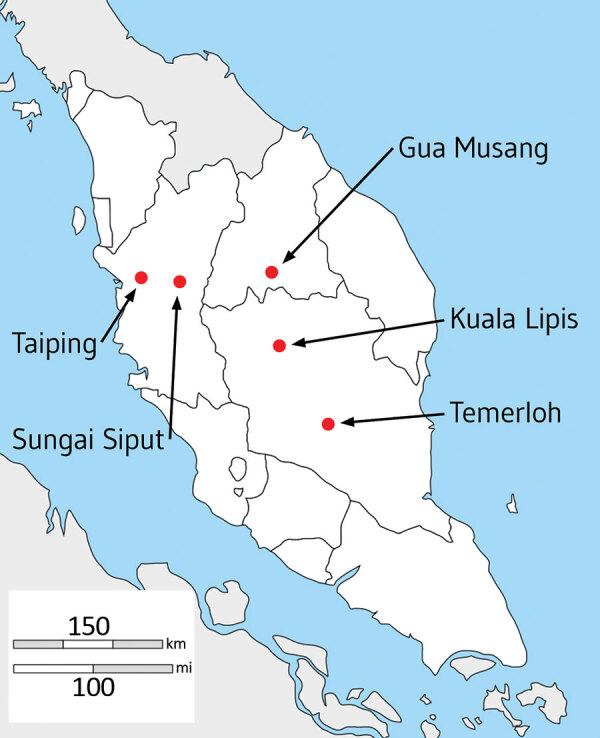
Locations of hospitals in peninsular Malaysia from which clinical *Plasmodium knowlesi* infections were sampled and sequenced in the states of Perak (Taiping and Sungai Siput), Kelantan (Gua Musang), and Pahang (Kuala Lipis and Temerloh). Of 56 infection samples processed through leukocyte depletion and subsequent DNA extraction, 32 had sufficient quantity and purity of *P. knowlesi* DNA for Illumina sequencing (https://www.illumina.com), of which 28 yielded high coverage genomewide sequence for population genomic analysis (sample and sequencing details listed in Appendix 1 Table).

We collected heparinized venous blood samples of up to 10 mL from 56 patients with *P. knowlesi* malaria at 5 hospitals in Peninsular Malaysia during February 2016–January 2018 (Figure 1; Appendix 1 Table), after obtaining written informed consent from each patient. The study was approved by the Medical Research and Ethics Committee of the Malaysia Ministry of Health and by the Ethics Committee of the London School of Hygiene and Tropical Medicine. 

We depleted leukocytes by passing each blood sample through a CF11 cellulose column to increase the proportion of parasite compared with host DNA. We extracted genomic DNA by using QIAamp DNA Mini kits (QIAGEN, https://www.qiagen.com) and confirmed that all contained only *P. knowlesi* by using nested PCR assays, testing for all locally known malaria parasite species (*22*). We lyophilized genomic DNA before transport to the United Kingdom, then dissolved the DNA in 30 μL of nuclease-free water and quantified it on a spectrophotometer by using the Quant-iT PicoGreen dsDNA Assay Kit (Thermo Fisher Scientific, https://www.thermofisher.com). We processed samples containing >300 ng of DNA for sequencing. We performed paired-end short-read genome sequencing by using Illumina MiSeq version 3 kits on the MiSeq platform (Illumina, https://www.illumina.com) with a read length of 300 bp, and aligned reads to the *P. knowlesi* PKNH 2.0 reference genome sequence (Appendix 1). After assembly and quality filtering, data from 28 clinical case samples were available for downstream analysis, representing samples from 5 hospitals in Peninsular Malaysia (Figure 1). For comparison with samples from elsewhere, we retrieved Illumina short reads from previous studies (*15*,*16*,*23*) and assembled them by using the identical pipeline as we had for the newly sequenced samples.

We masked from analysis parts of the genome to which short reads are difficult to uniquely map, including the subtelomeres and the multicopy *kir* and *SICAvar* gene families (Appendix 1). We called single-nucleotide polymorphisms (SNPs) by using a full repertoire of *P. knowlesi* genome sequences, including the 28 new sequenced infection samples from Peninsular Malaysia with high read coverage obtained in this study, as well as 74 previous samples from Malaysian Borneo (40 from cluster 1 and 34 from cluster 2) and 5 laboratory isolates (107 in total) (*15*,*16*,*23*). The procedures and parameters we used are comparable to those used for other original studies of *P. knowlesi* conducted previously (*15*,*16*) and to those used in population studies on endemic human malaria parasites, such as *P. falciparum*, that have much less diversity than *P. knowlesi* (*15*,*24*).

We used the packages adegenet (https://github.com/thibautjombart/adegenet/wiki) (*25*) and pegas (https://cran.r-project.org/web/packages/pegas/index.html) (*26*) in the R statistical framework to conduct principal component analysis and generate neighbor-joining trees by using an SNP-based pairwise genetic distance matrix. For population structure analysis, we used the package PopGenome (*27*) to calculate nucleotide diversity, within-population Tajima’s D indices, and between-population fixation indices (*F*_ST_). For sliding-window analysis genomewide, we calculated nucleotide diversity in nonoverlapping 50-kb windows. To scan for genes that might be under balancing selection, we calculated Tajima’s D on a gene-by-gene basis and excluded genes with <3 SNPs from analysis. We calculated *F*_ST_ values between each of the major parasite clusters for all individual SNPs across the genome with a minor allele frequency of >10%, and we calculated mean values in all nonoverlapping sliding windows of 500 consecutive SNPs across the genome.

We performed a scan for loci under recent positive selection by identifying SNPs with an allele associated with extended haplotype homozygosity, using the R package rehh (*28*) and applying the default setting assumption that common alleles are more likely to be ancestral. We calculated the standardized integrated haplotype score (|iHS|) for biallelic SNPs with no missing calls and with a minor allele frequency of >10%. We set SNPs with |iHS| values in the top 0.01% as core SNPs, around which we identified putative windows of selection by using the extended haplotype homozygosity (EHH) score, plotted until the EHH signal declined to <0.05 on each side. We merged overlapping windows of EHH containing some of the same SNPs to produce a contiguous overall putative selection window for that region, and we considered any gaps of >20 kb between SNPs with elevated |iHS| values to break a putative window of selection.

## Results

### Genomic Diversity of Different *P. knowlesi* Subpopulations

We successfully obtained paired-end short read Illumina genome sequences with high-read depth mapping to the *P. knowlesi* reference sequence from 28 *P. knowlesi* clinical case samples (Appendix 1) gathered from hospitals at 5 locations in Peninsular Malaysia (Figure 1). Analysis of these new infection samples together with previous sequences from infections in Malaysian Borneo enabled 994,761 SNPs to be initially called, of which 40,934 SNPs were removed because they were in genomic regions with generally unreliable short-read mapping (*kir* and *SICAvar* genes and subtelomeres), resulting in a total of 953,827 SNPs throughout the rest of the genome. After filtering out SNPs that had missing data in >10% of individual infection samples, we included 474,109 high-quality SNPs in subsequent analysis (Appendix 2 Datasheet 1).

**Figure 2:**
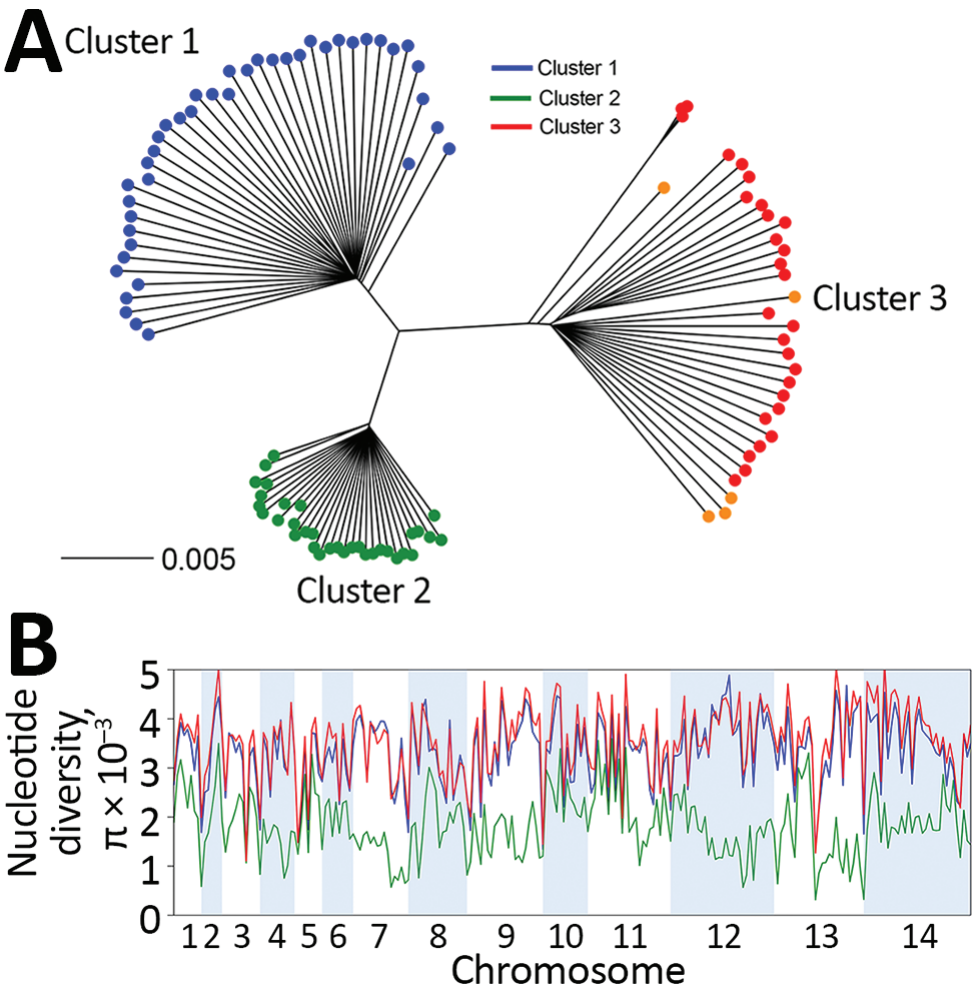
Genomewide analysis of diversity in *Plasmodium knowlesi* clinical samples from Peninsular Malaysia compared with samples from elsewhere. A) Neighbor-joining tree based on a pairwise genetic distance matrix between individual *P. knowlesi* infection samples for the 28 new clinical samples from Peninsular Malaysia (shown in red), 5 previously sequenced laboratory isolates (shown in orange), most of which were originally isolated from Peninsular Malaysia many years ago (*15*), and 74 samples from Malaysian Borneo that grouped into separate subpopulation clusters (cluster 1 shown in blue, cluster 2 in green) (*15*,*16*,*23*). All the clinical isolate samples from Peninsular Malaysia grouped into cluster 3 together with the laboratory isolates. The distance matrix is based on the proportion of all single-nucleotide polymorphisms (SNPs) showing differences between each infection sample (scale bar shows branch length for 0.5% of SNPs differing); most reads within each infection sample determine the allele scored for each SNP. B) Genomewide scan of nucleotide diversity (π) for *P. knowlesi* among the clinical isolates in Peninsular Malaysia (cluster 3, shown in red), compared with diversity observed in the subpopulations in Malaysian Borneo (clusters 1 and 2). The sliding window plot shows values of nucleotide diversity for nonoverlapping windows of 50 kb in each of the 14 chromosomes.

We generated a neighbor-joining tree by using pairwise genetic distances among individual *P. knowlesi* infection samples, which considered most nucleotide calls for all SNPs within each infection sample (Figure 2, panel A). The tree showed that all of the 28 samples from Peninsular Malaysia belonged to a genetic population (cluster 3) divergent from those in Malaysian Borneo (clusters 1 and 2). These new clinical samples from Peninsular Malaysia clustered with the old laboratory isolates (mostly from Peninsular Malaysia) that were sequenced previously and had initially indicated the existence of a third major genetic population within this species (*15*). The overall genomewide nucleotide diversity (π) among the new cluster 3 samples was 4.13 × 10^−3^ (the allele frequency spectrum is shown in Appendix 1 Figure 1), broadly similar to that for cluster 1 and higher than for cluster 2 (Figure 2, panel B). Differences between clusters 1 and 2 have been examined in detail in original analysis of parasites from Malaysian Borneo (*16*), and higher population genomic diversity within *P. knowlesi* has been noted in comparison with endemic malaria parasite species *P. vivax* and *P. falciparum* (*15*).

### Low Levels of Diversity within Individual Clinical Infections

**Figure 3:**
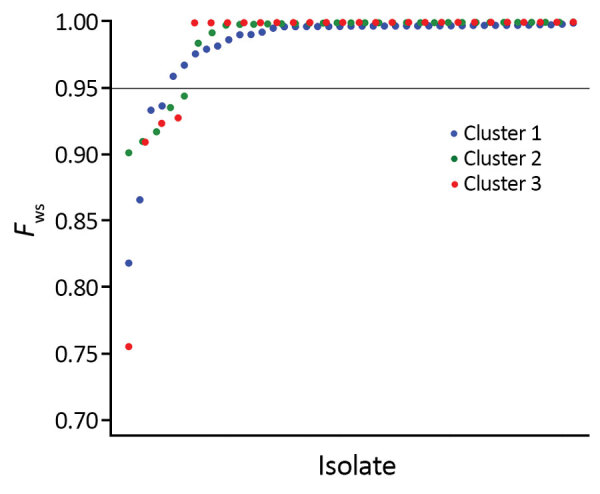
Low levels of diversity within individual *Plasmodium knowlesi* clinical infections from Malaysia as indicated by the high values of the genomewide within-isolate fixation index *F*_WS_ (potential range 0–1). A value of >0.95 is generally taken to indicate an infection dominated by a single genotype, whereas values <0.95 indicate infections that are clearly genotypically mixed. Each point shows the value for an individual infection sample; only 4 of the 28 cluster 3 clinical isolates from Peninsular Malaysia are clearly mixed (similar to the proportions observed in infections with the cluster 1 and 2 types in Malaysian Borneo).

Although all of the *P. knowlesi* clinical isolates were genotypically distinct from each other as indicated by considerable pairwise differences, most of them contained minimal within-infection sequence diversity, as indicated by the high values of the genomewide within-isolate fixation index *F*_WS_ (Figure 3). Of the 28 cluster 3 clinical isolates from Peninsular Malaysia in our study, only 4 were clearly mixed (with *F*_WS_ values <0.95). This low proportion of mixed genotype infections was similar to that observed for the different *P. knowlesi* subpopulations (clusters 1 and 2) in Malaysian Borneo (Figure 3).

### Population Genetic Substructure of *P. knowlesi* in Peninsular Malaysia

**Figure 4:**
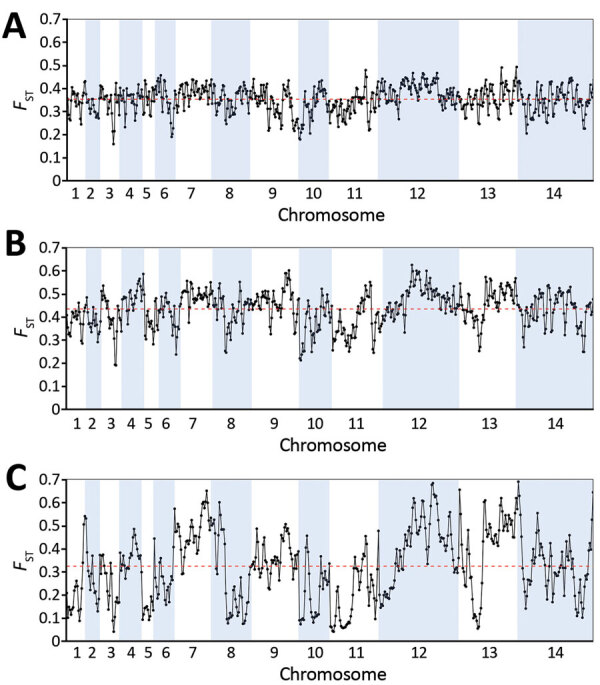
Genomewide between-population fixation index (*F*_ST_) scan of divergence between *Plasmodium knowlesi* in Peninsular Malaysia sampled in this study (cluster 3) and the major subpopulations previously sampled in Malaysian Borneo (clusters 1 and 2). All single-nucleotide polymorphisms (SNPs) with overall allele frequencies >10% were included, and the solid points show values for analysis windows containing 500 consecutive SNPs, centered by the midpoint of each sequential window and overlapping by 250 SNPs. The red dashed line on each plot shows the genomewide mean value for all analyzed SNPs across the genome. A) The level of divergence between cluster 3 in Peninsular Malaysia and cluster 1 in Malaysian Borneo does not differ greatly throughout the genome (mean *F*_ST_ value 0.32). B) Divergence between cluster 3 in Peninsular Malaysia and cluster 2 in Malaysian Borneo is slightly higher (mean *F*_ST_ value 0.42) and shows more heterogeneity between genomic regions because of mosaic structure of diversity in cluster 2 (as explained by panel C). C) Divergence between clusters 1 and 2 in Malaysian Borneo, showing marked heterogeneity across the genome that explains most of the moderate heterogeneity shown in panel B, attributable to a mosaic structure of diversity within cluster 2, as previously reported (*16*).

Analysis of SNP allele frequencies genomewide confirmed that the *P. knowlesi* cluster 3 population in Peninsular Malaysia is highly divergent from each of the separate clusters 1 and 2 in Malaysian Borneo (Figure 4). Comparison of cluster 3 with cluster 1 reveals a genomewide mean *F*_ST_ values of 0.32 (with 3,713 SNPs being at complete fixation), whereas comparison of cluster 3 with cluster 2 reveals a genomewide mean *F*_ST_ value of 0.42, (with 6,738 SNPs being at complete fixation) (Figure 4; Appendix 1 Figure 2). We observed a high consistency across the genome in the level of divergence when comparing cluster 3 with cluster 1 (Figure 4, panel A), but more variation across the genome was apparent in the comparison between cluster 3 and cluster 2 (Figure 4, panel B). This finding is attributable to a previously described mosaic pattern of diversity across the genome of cluster 2 (*16*), which contributes to greater genomewide heterogeneity in divergence between clusters 1 and 2 in Malaysian Borneo (Figure 4, panel C) than between either of these and cluster 3 in Peninsular Malaysia.

**Figure 5:**
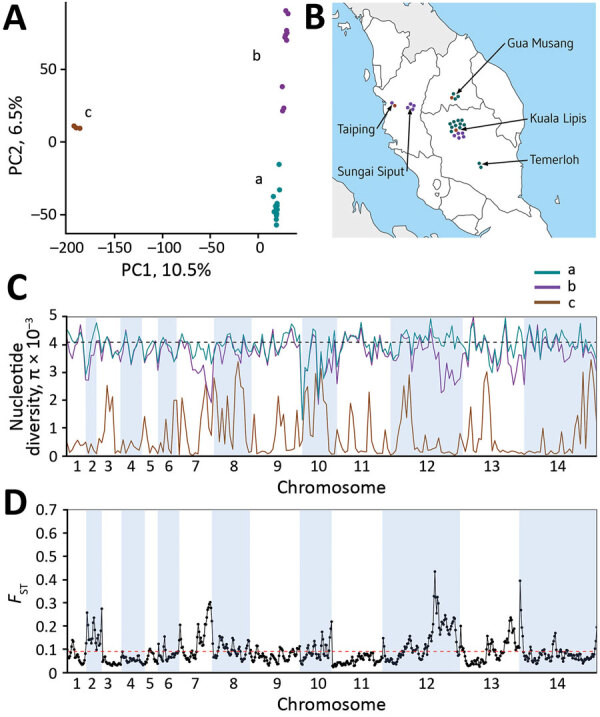
*Plasmodium knowlesi* cluster 3 clinical isolates forming genomic subpopulations that co-occur locally, Peninsular Malaysia. A) Principal component analysis of the 28 cluster 3 *P. knowlesi* clinical isolates from Peninsular Malaysia, showing clustering into 3 groups: subclusters A (15 isolates), B (10 isolates), and C (3 isolates). The assignment of all samples to these 3 subclusters is completely consistent with their placement in the within-cluster 3 branching of the neighbor-joining tree based on the pairwise distance matrix (Figure 2, panel A). The first principal component accounts for 10.5% of overall variation and separated subcluster 3 from the others, whereas the second principal component accounts for 6.5% of overall variation and separated subclusters A and B. B). Each of the cluster 3 *P. knowlesi* subclusters was detected at multiple sites within peninsular Malaysia (points shown at each of the 5 sampling sites show individual infections with colors for the different subclusters as in panel A). The site with most samples had all 3 subclusters co-occurring locally. C) Genomewide scan of diversity shows that the subcluster C samples are virtually identical in large parts of the genome, whereas subclusters A and B are both highly diverse throughout the genome, with only a few genomic regions showing lower diversity in subcluster B compared with A (in chromosomes 2, 7, 12, and 13). D) Genomewide scan of differentiation between subclusters A and B by sliding window between-population fixation index analysis shows peaks of differentiation corresponding to regions with differences in diversity. Most notable is a large region of chromosome 12 having many windows with between-population fixation index values >0.2 and containing some individual single-nucleotide polymorphisms with fixed differences (Appendix 1 Figure 2).

The distance matrix-based neighbor-joining tree indicated internal branching of the *P. knowlesi* cluster 3 clinical samples from Peninsular Malaysia into 3 different subclusters (Figure 2, panel A). To examine this branching further, we focused the principal component analysis on the clinical samples from Peninsular Malaysia alone, which showed that they formed 3 groups (Figure 5, panel A). The smallest group contained 3 of the samples (GMK03, TPK03, and KLK12) separated from the rest along principal component 1 (which explained 10.5% of overall variation), whereas the 2 other groups were less tightly separated along principal component 2 (which explained 6.5% of overall variation) (Figure 5, panel A). These 3 groups existing within *P. knowlesi* cluster 3 in Peninsular Malaysia are considered as subpopulations, designated in this article as subclusters A (15 of the infections), B (10 infections), and C (3 infections), which are also apparent in neighbor-joining analysis of the cluster 3 samples alone (Appendix 1 Figure 3). These different parasite genetic subclusters are not separated geographically within Peninsular Malaysia, each being detected from multiple sites (Figure 5, panel B), and the hospital with the largest sample size (in Kuala Lipis) had infections of all 3 subclusters. Moreover, infections of the different subcluster types were not temporally aggregated (Appendix 1 Table).

The most divergent of these types (subcluster C) consisted of infections that were highly related to each other, virtually identical in many parts of the genome (Figure 5, panel C). This finding is remarkable because each of these cases were sampled from different states within Peninsular Malaysia (Figure 4, panel B). Although subclusters A and B had similar levels of nucleotide diversity to each other, sliding-window analysis indicated a few genomic regions in which subcluster B has lower diversity than subcluster A (e.g., in a region covering half of chromosome 12) (Figure 5, panel C). Genomewide scan of differentiation between subclusters A and B by sliding-window *F*_ST_ analysis showed peaks of high differentiation against a background of low differentiation in most of the genome (Figure 5, panel D). The regions that showed differences in levels of diversity are also the most differentiated between the subclusters, most notably the large region of chromosome 12, which has many windows with *F*_ST_ values exceeding 0.2 (Figure 5, panel D) and contains some individual SNPs with fixed differences (Appendix 1 Figure 4).

### Identification of Genomic Loci under Recent Selection in Peninsular Malaysia

**Figure 6:**
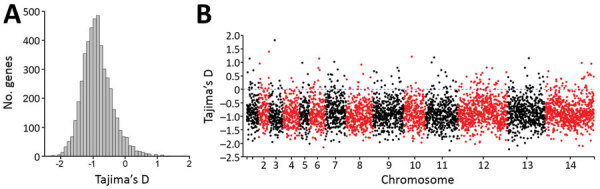
Summary of nucleotide site allele frequency distributions by Tajima’s D indices for all 4,742 *Plasmodium knowlesi* genes with >3 SNPs among the 28 cluster 3 *P. knowlesi* infections in Peninsular Malaysia. A) Overall values were negatively skewed with a mean Tajima’s D of −0.86, consistent with a pattern that would be caused by long-term population size expansion. B) Data for all individual genes show that those with high Tajima’s D values are distributed throughout the genome. Some of these genes are likely to be underbalancing selection (individual values for all genes are shown in Appendix 2 Datasheet 2).

To scan for loci that might be under different selection pressures in *P. knowlesi* in Peninsular Malaysia, we summarized nucleotide site allele frequency spectra by calculating Tajima’s D index for all 4,742 genes with >3 SNPs. Overall, values were negatively skewed (mean −0.86) (Figure 6, panel A); only 215 genes had values >0, of which only 8 had values >1.0 (Figure 6, panel B). This genomewide pattern is consistent with expectations if long-term population size expansion had occurred. Individual genes with unusually high Tajima’s D values (Figure 6, panel B) might be under balancing selection and might be examined separately (Appendix 2 Datasheet 2). Some genes with high values have orthologs in other malaria parasite species that are likely targets of immunity, including a tryptophan-rich protein (PKNH_1472400, D = 0.98), a 6-cysteine protein (PKNH_1254400, D = 0.61), an exported protein PHIST (PKNH_0808500, D = 0.57), and an MSP7-like protein (PKNH_1265900, D = 1.15). However, we found that some other genes with orthologs considered to be targets of immunity in other malaria parasite populations had negative Tajima’s D values, including the circumsporozoite protein (*csp*) gene, which had the highest Tajima’s D value genomewide in cluster 1 *P. knowlesi* in Malaysian Borneo (*15*), as well as the apical membrane antigen 1 gene (*ama1*, D = −1.35), the Duffy binding protein α (*DBPα*, D = −0.89), and the normocyte binding protein gene (NBPXa, D = −0.42). These findings indicate that the mode or strength of selection on orthologous targets is not uniform in all malaria parasite populations, including among different *P. knowlesi* subpopulations.

**Figure 7:**
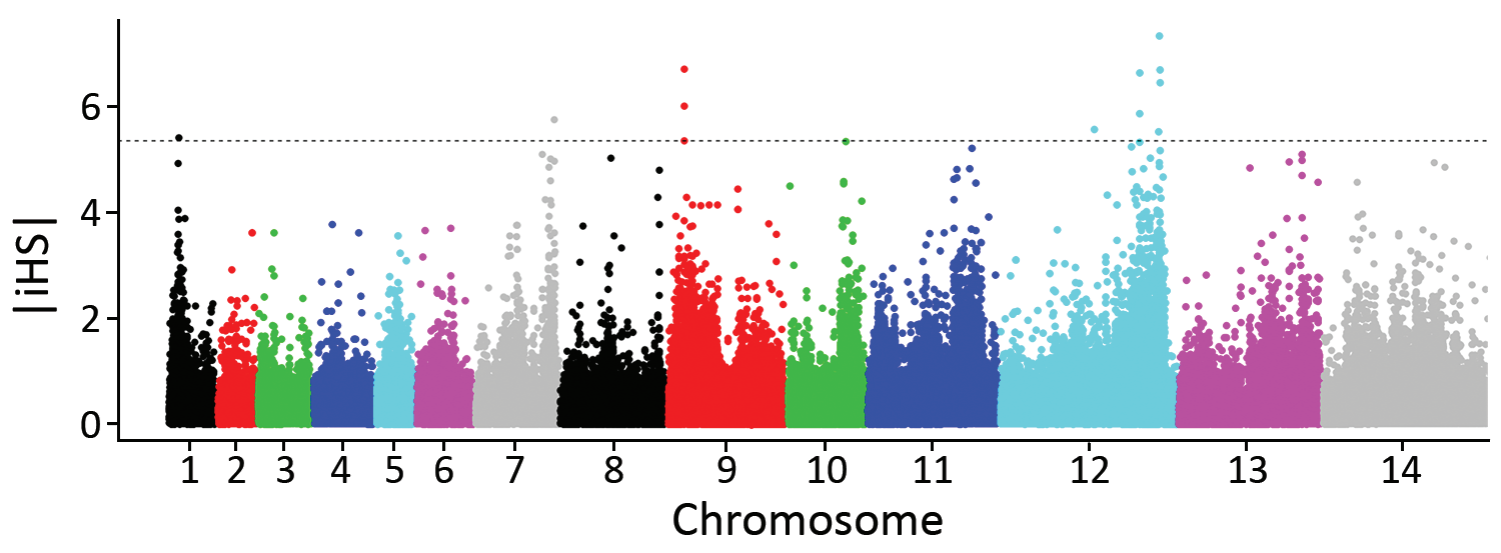
Scan for evidence of genomic regions affected by recent positive directional selection in *Plasmodium knowlesi* in Peninsular Malaysia, using the standardized integrated haplotype score |iHS| index. Examination of the ranges of extended haplotype homozygosity for individual single-nucleotide polymorphisms (SNPs) with high |iHS| values identified 4 distinct genomic windows of extended haplotypes (Appendix 2 Datasheet 3). Two of these (in chromosomes 1 and 9) spanned across *SICAvar* and *kir* genes, which were masked from SNP calling, whereas the other 2 did not include *SICAvar* or *kir* genes but covered ≈28 kb on chromosome 7 and ≈315 kb on chromosome 12. The large region on chromosome 12 is a merged window, consisting of 4 high |iHS| core SNPs with overlapping windows of extended haplotype homozygosity, and coincides with the region of chromosome 12 that has the highest genomic divergence between cluster 3 population subclusters A and B (Figure 5, panel D).

We used the standardized integrated haplotype score |iHS| index as a means of scanning for evidence of genomic regions that are likely to have been affected by recent positive directional selection. Analyzing the full population sample of clinical isolates from Peninsular Malaysia, we observed that 11 SNPs had standardized |iHS| values in the top 0.01%, and examination of the ranges of their extended haplotype homozygosity identified 4 distinct genomic windows of extended haplotypes (Figure 7; Appendix 2 Datasheet 3). Two of these (in chromosomes 1 and 9) spanned across genomic *SICAvar* and *kir* genes that had been masked from SNP calling and analysis. The other 2 windows of extended haplotype homozygosity did not include *SICAvar* or *kir* genes but covered ≈28 kb (11 genes) on chromosome 9 and ≈315 kb (81 genes) on chromosome 12. The large region of elevated |iHS| values on chromosome 12 coincides with the region having the highest genomic divergence between cluster 3 population subclusters A and B (Figure 5, panel D), indicating that recent selection on this region has affected part of the *P. knowlesi* population and contributed to the local genetic substructure in Peninsular Malaysia.

## Discussion

Genomewide sequence analysis of new clinical isolates has revealed unexpected parasite population structure and evidence of recent selection in *P. knowlesi* in Peninsular Malaysia. On the basis of previous multilocus microsatellite analysis, we expected that genome sequencing of samples from Peninsular Malaysia would reveal a parasite population distinct from those previously described in Malaysian Borneo. This distinction was indeed clearly shown; all samples from Peninsular Malaysia belonged to a genetic population (cluster 3) that is highly divergent genomewide from both of the clusters 1 and 2 in Malaysian Borneo (mean *F*_ST_ values of 0.32 for cluster 1 and 0.42 for cluster 2). However, the cluster 3 clinical samples from Peninsular Malaysia themselves constituted 3 distinct subpopulations, and the cause of this local population genetic structure needs to be determined. No geographic separation is apparent; each of the three cluster 3 subpopulations was found in overlapping locations, and all were detected from among cases in the hospital that had most samples analyzed. The population structure might reflect >1 local zoonotic transmission cycle or could be a sign of recent selection and emergence of a subpopulation of *P. knowlesi* transmitted more effectively between humans.

In Malaysian Borneo, long-tailed macaques are reservoir hosts for the cluster 1 population of *P. knowlesi*, whereas pig-tailed macaques are reservoir hosts for the cluster 2 population (*13*,*14*), but whether different reservoir hosts contribute to the parasite population structure we have shown within Peninsular Malaysia is unknown. Microsatellite analysis of *P. knowlesi* in long-tailed macaques from Peninsular Malaysia has indicated that most of them belong to cluster 3, although some samples from long-tailed macaques had indeterminate cluster assignments (*13*). Our findings underscore the need to sample and genotype parasites from pig-tailed macaques in Peninsular Malaysia, as well as to analyze more samples from long-tailed macaques, to investigate whether the parasite subclusters have different reservoir host species locally.

Genetic subpopulations of *P. knowlesi* in Peninsular Malaysia might also be transmitted by different mosquito species. *P. knowlesi* is transmitted by the *Anopheles* Leucosphyrus group of mosquitoes, which contains a diverse array of species found throughout Southeast Asia (*29*), including *An. latens*, *An. cracens, An. introlatus*, and *An. hackeri*, in which *P. knowlesi* has been detected in Peninsular Malaysia, as well as other species that have been shown to be infected elsewhere (*30*). *Anopheles* Leucosphyrus group mosquitoes predominantly inhabit forested areas (*31*,*32*), so changes to forest areas and ongoing deforestation will affect human exposure. The potential vector species vary in relative abundance among different sampling sites in Peninsular Malaysia (*33*–*35*), but more surveys are required to determine the relative extent to which they transmit *P. knowlesi* and whether they transmit different populations of the parasite (*36*).

Genomewide scanning revealed discrete regions of divergence between subclusters A and B of *P. knowlesi* cluster 3, in particular a large region on chromosome 12. Interestingly, this region had the strongest evidence of recent directional selection, as indicated by the integrated haplotype score analysis. Moreover, this genomic region did not show evidence of recent selection in Malaysian Borneo (*15*), so the signature is specific to Peninsular Malaysia and indicates selection to be operating locally.

Even more unexpected is the observation of a separate *P. knowlesi* cluster 3 subpopulation (subcluster C), represented by 3 infections highly related to each other throughout most of the genome. Although less common, clinical cases with this parasite type were identified in different hospitals in 3 different states in Peninsular Malaysia. Population genetic substructure also has been observed in the endemic malaria parasites *P. falciparum* (*37*) and *P. vivax* (*38*) in Malaysia, although that observation has been interpreted as indicating fragmented populations that are close to being eliminated. Notable substructure of *P. falciparum* populations also has been observed in Cambodia, probably because of strong selection on locally emerging drug-resistant types in areas where transmission was low (*39*). Zoonotic *P. knowlesi* populations are probably substructured for other reasons, as previously observed in Malaysian Borneo, where the 2 divergent parasite genetic populations observed in human cases are associated with different reservoir host species (*13*,*14*).

Population genomic analysis of *P. knowlesi* so far has mainly focused on parasites from Malaysia, where most reported cases of *P. knowlesi* malaria have been identified. However, cases of *P. knowlesi* malaria in humans have now been reported from all Southeast Asia countries. Whether other local zoonotic subpopulations exist throughout the region or whether all parasites belong to the major genetic populations observed in Malaysia is unknown. Our findings highlight the importance of monitoring population genetic changes in Malaysia and conducting comparable analysis in other areas where *P. knowlesi* has only very recently been realized to occur in humans (*40*,*41*).

Appendix 1 Additional information about population genomic structure and recent evolution of *Plasmodium knowlesi*, peninsular Malaysia.

Appendix 2 Additional data for population genomic structure and recent evolution of *Plasmodium knowlesi*, peninsular Malaysia.
