# Supplementary material for: Population Genomic Structure and Recent Evolution of Plasmodium knowlesi, Peninsular Malaysia
Source: Emerg Infect Dis. 2020 Aug;26(8):1749–58. doi: 10.3201/eid2608.190864 (PMC7392424; doi:10.3201/eid2608.190864)
Supplement: Appendix 1 — Additional information about population genomic structure and recent evolution of Plasmodium knowlesi, peninsular Malaysia. [file 19-0864-Techapp-s1.pdf]

Article DOI: https://doi.org/10.3201/eid2608.190864

Population Genomic Structure and Recent Evolution of *Plasmodium knowlesi*, Peninsular Malaysia

Appendix 1

Sequencing and SNP Calling Methods

Preparation of libraries for Illumina paired-end short read genome sequencing was performed using 300ng of input DNA from each patient sample with the TruSeq Nano DNA kit (Illumina), with genomic DNA first being sheared into fragments with average size of 550 bp using an M220-Focused Ultrasonicator (Covaris). Library quantification was carried out by quantitative PCR (qPCR) using the KAPA Library Quantification kit for Illumina (KAPA Biosystems) and library quality was assessed with the Agilent High Sensitivity DNA kit on the Agilent Bioanalyser (Agilent). Any samples showing significant primer dimer content or containing less than 4nM of DNA by qPCR were not processed for sequencing. Samples passing quality criteria were normalized to 4nM and pooled equimolar in batches of no more than eight. Library pools were denatured and diluted to a final concentration of 15pM and spiked with 1% PhiX.

Paired-end sequencing was performed on an Illumina MiSeq using 600-cycle v3 MiSeq reagents with a read length of 300 bp, and raw data were generated in FASTQ format, with FastQC (http://www.bioinformatics.babraham.ac.uk/projects/fastqc/) used to assess quality of the short reads and inform the trimming to remove low quality base calls. Numbers of short reads per sample ranged from 3.2 million to 9.8 million, and the GC content of each sample was checked, so that any straying from the expected 38% GC content of the *Plasmodium knowlesi* genome was inspected in case of potential contamination.

Short reads were first trimmed with Trimmomatic v0.3.2 using default parameters (*1*), and then aligned to the *P. knowlesi* PKNH version 2.0 reference genome with BWA-MEM (*2*). Mapped reads were converted to bam format, sorted, and indexed using samtools version 1.3.1

(*3*). Reads originating from PCR duplicates were removed using Picard (https://broadinstitute.github.io/picard). Average read depth was calculated using SAMtools and samples with an average coverage of less than 20x were excluded from further analysis.

The first round of SNP calling was performed on each sample independently. SNPs were initially marked using samtools mileup run with the following flags: *-B -I -Q 23 -d 2000 -C 50*, and marked SNPs were then called using *bcftools call -m -v* and filtered using *vcfutils.pl varFilter -d 10 -D 2000*. The resulting per-sample SNP lists were then filtered, and positions with a read depth of less than 30x were discarded. The SNPs for each sample were then compiled into a single file, resulting in a 'unique SNP list' representing the *P. knowlesi* population, containing SNP positions and alternate bases. Any SNPs falling within subtelomeric regions or *kir* and *SICAvar* gene regions as noted above were filtered out of the unique SNP lists using bedtools intersect v2.27.0 (*4*). A second round of SNP calling and filtering was then implemented using custom made Perl scripts (*5*) to make the major genotype call at each SNP position for each sample. SNP positions that had 50:50 split in mixed base calls for a particular sample were uncalled and scored as 'N'. Positions that had data missing in more than 10% of the infection samples were not analyzed.

Parts of the genome are difficult to uniquely map short reads to, and were therefore masked from analysis, including the subtelomeres and the multi-copy *kir* and *SICAvar* gene families and low complexity intergenic regions between these. Boundaries of subtelomeric regions were defined as in previous analysis (*6*), while *kir* and *SICAvar* genes were identified using the gene search function of PlasmoDB (www.plasmodb.org) and verified by inspecting the *P. knowlesi* annotation Embl files in Artemis (*7*). In genomic regions where several polymorphic genes belonging to *kir* and *SICAvar* families occurred sequentially, the masked region was extended to include all genes and intergenic sequence, and extended on each side until the first SNP called after the final masked gene in that region.

References

1. Bolger AM, Lohse M, Usadel B. Trimmomatic: a flexible trimmer for Illumina sequence data. Bioinformatics. 2014;30:2114–20. PubMed https://doi.org/10.1093/bioinformatics/btu170

2. Li H, Durbin R. Fast and accurate short read alignment with Burrows-Wheeler transform. Bioinformatics. 2009;25:1754–60. PubMed https://doi.org/10.1093/bioinformatics/btp324

3. Li H, Handsaker B, Wysoker A, Fennell T, Ruan J, Homer N, et al.; 1000 Genome Project Data Processing Subgroup. The Sequence Alignment/Map format and SAMtools. Bioinformatics. 2009;25:2078–9. PubMed https://doi.org/10.1093/bioinformatics/btp352

4. Quinlan AR, Hall IM. BEDTools: a flexible suite of utilities for comparing genomic features. Bioinformatics. 2010;26:841–2. PubMed https://doi.org/10.1093/bioinformatics/btq033

5. Assefa S, Lim C, Preston MD, Duffy CW, Nair MB, Adroub SA, et al. Population genomic structure and adaptation in the zoonotic malaria parasite *Plasmodium knowlesi.* Proc Natl Acad Sci U S A. 2015;112:13027–32. PubMed https://doi.org/10.1073/pnas.1509534112

6. Divis PCS, Duffy CW, Kadir KA, Singh B, Conway DJ. Genome-wide mosaicism in divergence between zoonotic malaria parasite subpopulations with separate sympatric transmission cycles. Mol Ecol. 2018;27:860–70. PubMed https://doi.org/10.1111/mec.14477

7. Rutherford K, Parkhill J, Crook J, Horsnell T, Rice P, Rajandream MA, et al. Artemis: sequence visualization and annotation. Bioinformatics. 2000;16:944–5. PubMed https://doi.org/10.1093/bioinformatics/16.10.944

Appendix 1 Table. Origins and primary data on the 28 *Plasmodium knowlesi* clinical infection samples from Peninsular Malaysia with genomewide sequences obtained in this study

Infection sample	Location	Date collected	Parasitemia μl^{-1} blood	Total reads (x 10^6)	Genomewide mean read depth coverage	ENA accession no.
GMK02	Gua Musang	2016 Nov 5	88,164	5.8	47x	ERS3517629
GMK03	Gua Musang	2017 Feb 19	16,480	5.6	40x	ERS3517630
GMK06	Gua Musang	2018 Jan 15	98,800	8.7	74x	ERS3517631
GMK07	Gua Musang	2018 Jan 21	12,900	7.9	66x	ERS3517632
KLK02	Kuala Lipis	2016 Jul 29	1,932	3.2	40x	ERS3517633
KLK04	Kuala Lipis	2016 Aug 9	50,938	4.0	32x	ERS3517634
KLK05	Kuala Lipis	2016 Sep 18	27,251	5.0	36x	ERS3517635
KLK06	Kuala Lipis	2016 Oct 23	661,875	5.8	43x	ERS3517636
KLK08	Kuala Lipis	2017 Jan 30	84,400	3.6	91x	ERS3517637
KLK12	Kuala Lipis	2017 Mar 2	7,236	3.6	27x	ERS3517638
KLK14	Kuala Lipis	2017 Apr 9	33,060	5.8	46x	ERS3517639
KLK15	Kuala Lipis	2017 Apr 12	136,408	5.4	37x	ERS3517640
KLK16	Kuala Lipis	2017 May 17	13,756	5.4	37x	ERS3517641
KLK17	Kuala Lipis	2017 May 19	22,554	5.8	44x	ERS3517642
KLK19	Kuala Lipis	2017 Jul 11	46,035	4.4	37x	ERS3517643
KLK21	Kuala Lipis	2017 Aug 28	60,950	4.5	39x	ERS3517644
KLK23	Kuala Lipis	2017 Feb 25	23,288	8.3	71x	ERS3517645
KLK24	Kuala Lipis	2017 Dec 7	141,069	5.7	47x	ERS3517646
KLK25	Kuala Lipis	2018 Jan 16	54,352	3.7	32x	ERS3517647
KLK27	Kuala Lipis	2018 Jan 19	69,160	7.8	64x	ERS3517648
SSK01	Sungai Siput	2016 Sep 30	6,027	8.8	40x	ERS3517649
SSK02	Sungai Siput	2016 Dec 2	2,763	7.6	62x	ERS3517650
SSK04	Sungai Siuit	2017 Jan 25	6,750	3.6	22x	ERS3517651
SSK05	Sungai Siput	2017 Mar 7	6,750	3.8	30x	ERS3517652
TK01	Temerloh	2016 Aug 2	3,806	4.4	21x	ERS3517653
TK03	Temerloh	2017 Feb 21	10,323	6.0	49x	ERS3517654
TPK03	Taiping	2017 Aug 30	4,000	9.8	55x	ERS3517655
TPK06	Taiping	2017 Nov 15	12,494	8.8	74x	ERS3517656

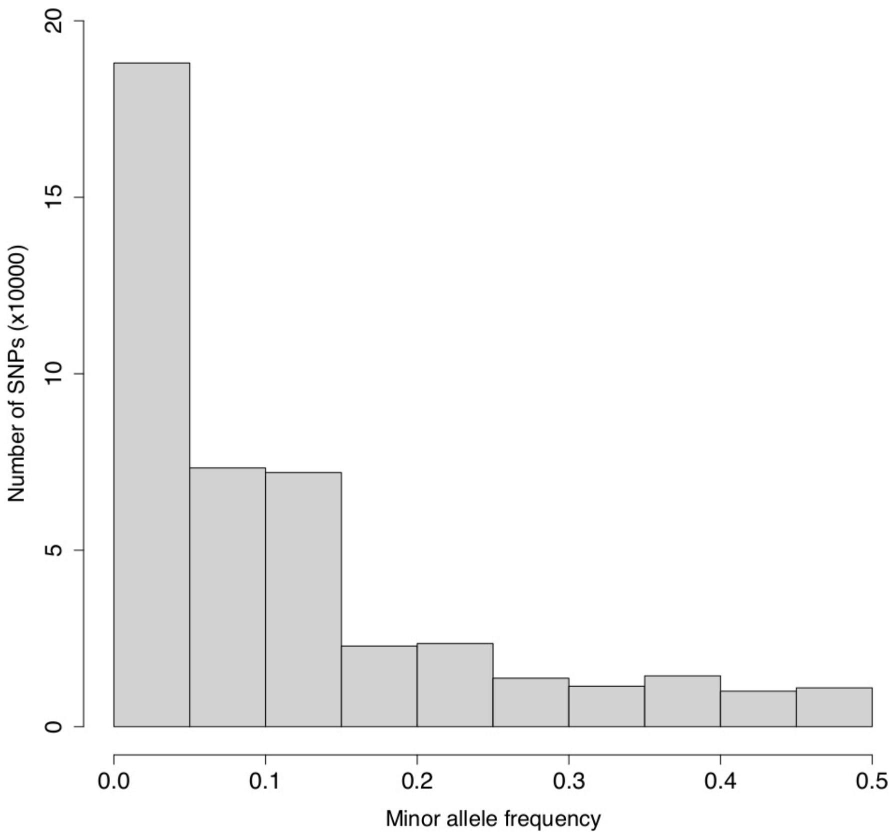

Appendix 1 Figure 1. Minor allele frequency distribution of all SNPs analyzed in *Plasmodium knowlesi* cluster 3 (sample of 28 clinical isolates from Peninsular Malaysia).

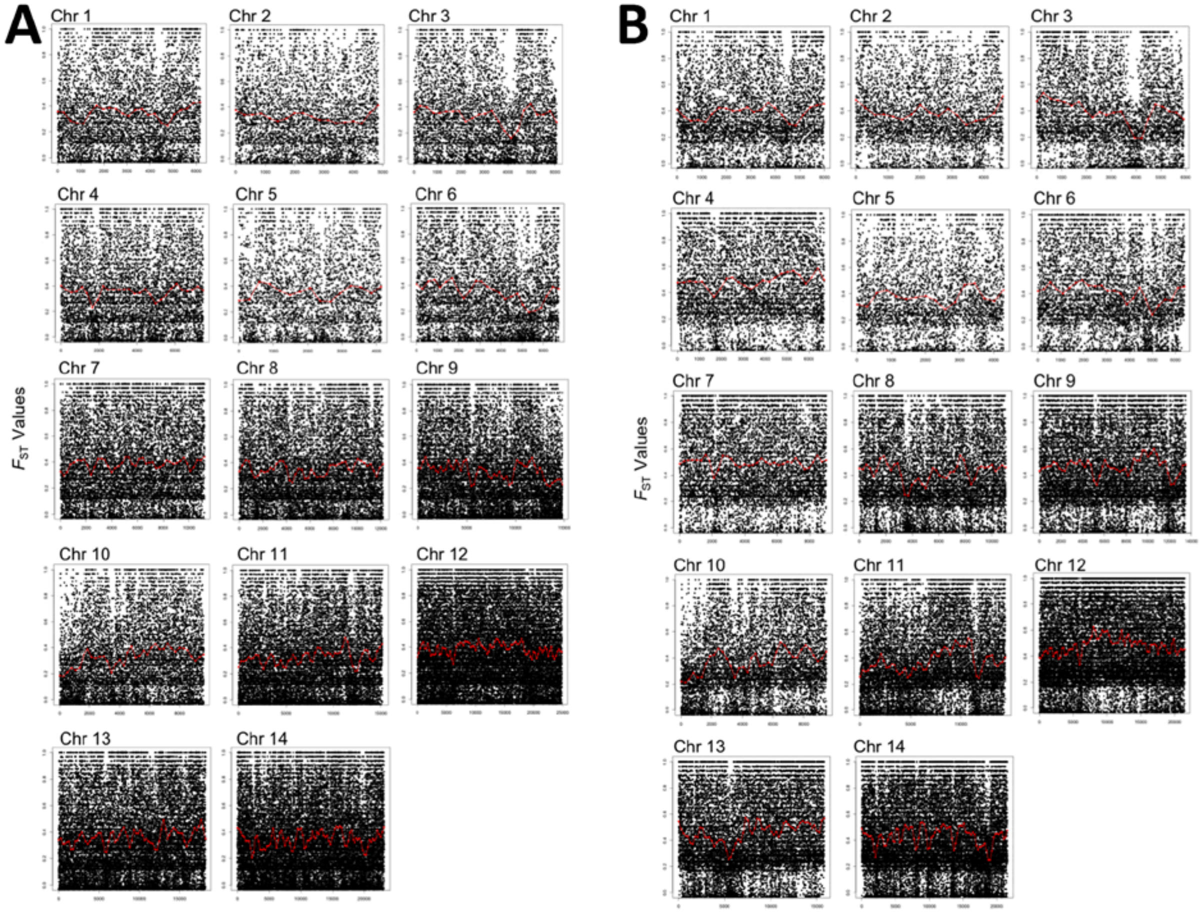

Appendix 1 Figure 2. Genomewide plots of F_{ST} values of allele frequency divergence for all individual SNPs, for comparisons between the *Plasmodium knowlesi* population in Peninsular Malaysia (cluster 3) and each of the two genetic populations in Malaysian Borneo (clusters 1 and 2). A. cluster 3 versus cluster 1. B. Cluster 3 versus cluster 2. Comparison between *P. knowlesi* cluster 1 and 2 is not shown as this was published previously (Divis PCS, Duffy CW, Kadir KA, Singh B, Conway DJ. Genomewide mosaicism in divergence between zoonotic malaria parasite subpopulations with separate sympatric transmission cycles. Mol Ecol. 2018;2:860–70). F_{ST} values shown for all individual SNPs with MAF >10%, comparing between *P. knowlesi* cluster 3 in Peninsular Malaysia and cluster 2 in Malaysian Borneo. In red are shown the means for overlapping windows of 500 SNPs (step size of 250 SNPs along chromosomes).

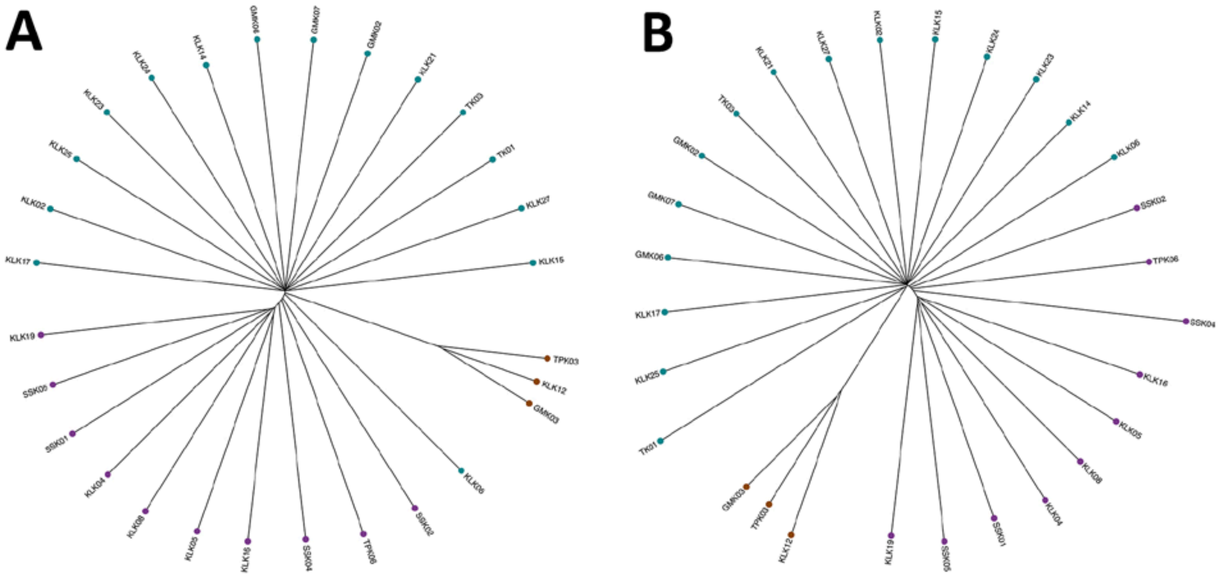

Appendix 1 Figure 3. Neighbor-Joining trees (NJ) indicating clustered relatedness among 28 new clinical isolates within *Plasmodium knowlesi* cluster 3. A. Distance matrix based on all SNPs genomewide. B. Distance matrix based on SNPs genomewide excluding those on chromosome 12.

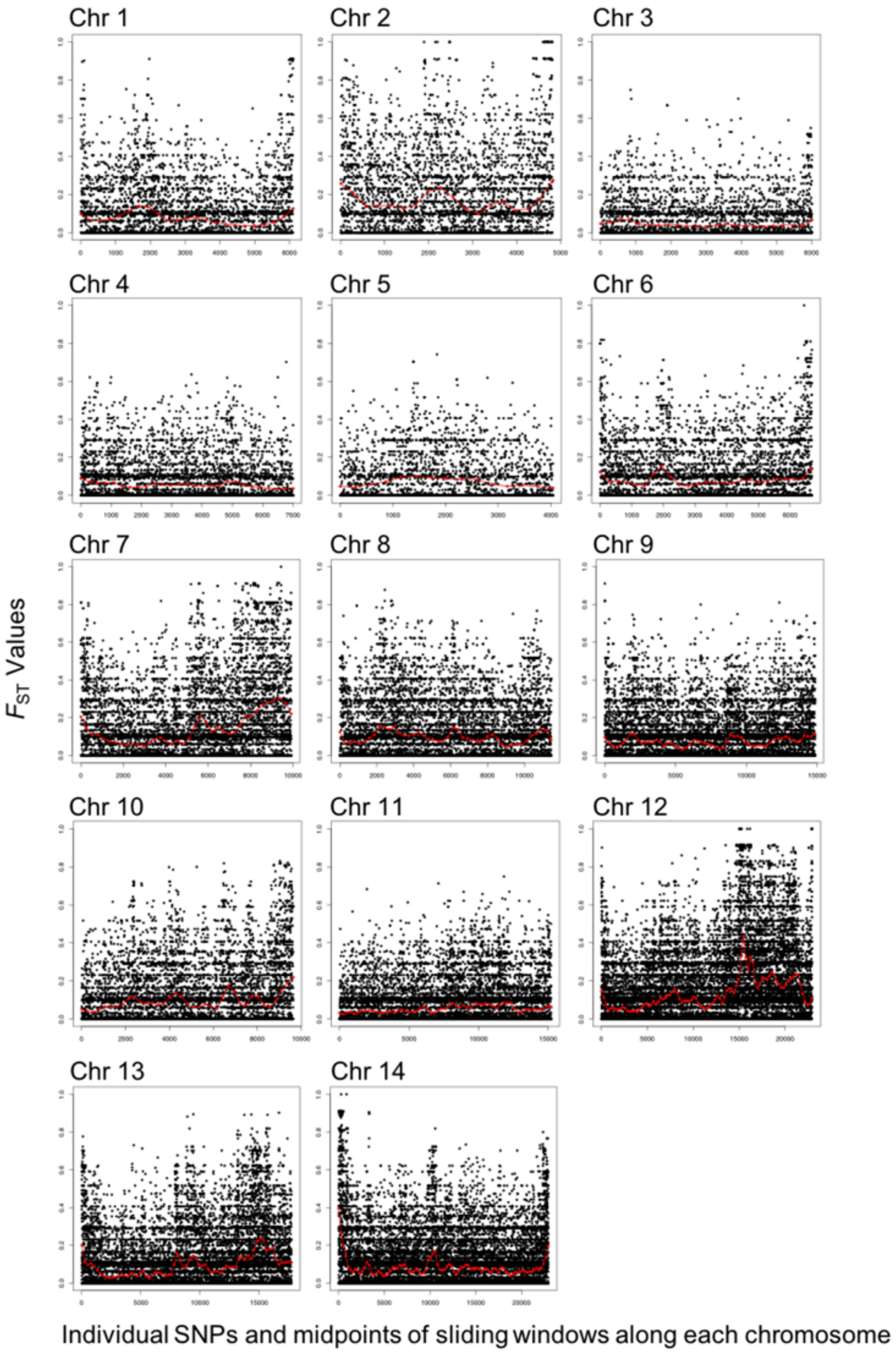

Appendix 1 Figure 4. Genomewide plots of F_{ST} values of allele frequency divergence for all individual SNPs, for comparisons between the two largest subpopulations of *Plasmodium knowlesi* cluster 3 (subclusters A and B) within the population in Peninsular Malaysia. F_{ST} values shown for all individual SNPs with MAF >10%, comparing between subclusters A and B of *P. knowlesi* cluster 3 within Peninsular Malaysia. In red are shown the means for overlapping windows of 500 SNPs (step size of 250 SNPs along chromosomes).
